# Age at diagnosis of diabetes in Appalachia

**DOI:** 10.1186/1478-7954-9-54

**Published:** 2011-09-30

**Authors:** Lawrence Barker, Robert Gerzoff, Richard Crespo, Molly Shrewsberry

**Affiliations:** 1 Division of Diabetes Translation, Centers for Disease Control and Prevention, Atlanta Georgia, USA; 2 Department of Family and Community Health, Marshall University, Huntington West Virginia, USA

**Keywords:** Appalachia, Diabetes: Disparities, Geography

## Abstract

**Background:**

Appalachia is a region of the United States noted for the poverty and poor health outcomes of its residents. Residents of the poorest Appalachian counties have a high prevalence of diabetes and risk factors (obesity, low income, low education, etc.) for type 2 diabetes. However, diabetes prevalence exceeds what these risk factors alone explain. Based on this, the history of poor health outcomes in Appalachia, and personally observed high rates of childhood obesity and lack of concern about prediabetes, we speculated that people in Appalachia with diagnosed diabetes might tend to be diagnosed younger than their non-Appalachian counterparts.

**Methods:**

We used data from the Behavioral Risk Factor Surveillance System (2006-2008). We compared age at diagnosis among counties by Appalachian Regional Commission-defined level of economic development. To account for risk differences, we constructed a model for average age at diagnosis of diabetes, adjusting for county economic development, obesity, income, sedentary lifestyle, and other covariates.

**Findings:**

After adjustment for risk factors for diabetes, people in distressed or at-risk counties (the least economically developed) had their diabetes diagnosed two to three years younger than comparable people in non-Appalachian counties. No significant differences between non-Appalachian counties and Appalachian counties at higher levels of economic development remained after adjusting.

**Conclusions:**

People in distressed and at-risk counties have poor access to care, and are unlikely to develop diabetes at the same age as their non-Appalachian counterparts but be diagnosed sooner. Therefore, people in distressed and at-risk counties are likely developing diabetes at younger ages. We recommend that steps to reduce health disparities between the poorest Appalachian counties and non-Appalachian counties be considered.

## Background

**Figure 1:**
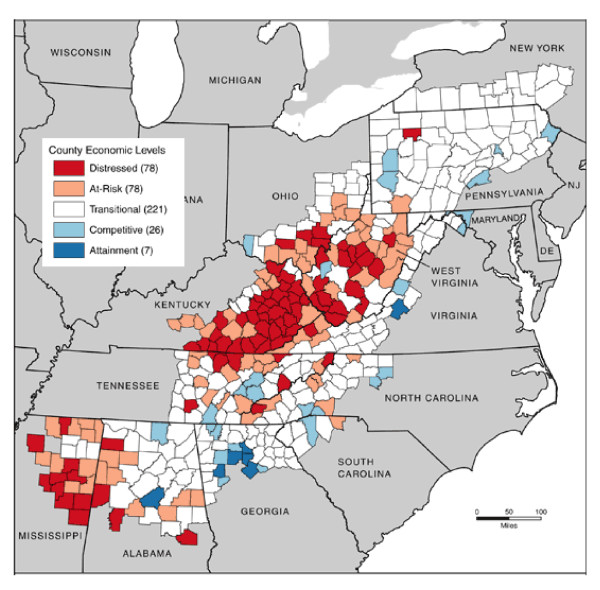
**Map of Appalachia showing county development level as of 2007**.

The Appalachian region of the United States extends from southern New York to northern Mississippi [1] (Figure 1). Appalachia includes all of West Virginia and parts of Alabama, Georgia, Kentucky, Maryland, Mississippi, New York, North Carolina, Ohio, Pennsylvania, South Carolina, Tennessee, and Virginia. Approximately 42% of Appalachia's population of 24 million people is rural, compared to 20% of the national population [1]. In 2000, Appalachia's population was 88% non-Hispanic white, as compared with about 70% for the rest of the United States [2].

Appalachia was slow to develop large urban centers, due in part to rough terrain and a shortage of roads and navigable rivers. In the early days of westward expansion, settlers of the mountainous, often steep-sloped terrain in parts of Appalachia found land adequate for their needs. However, as western land opened, Appalachia became increasingly economically marginalized. Instead of exhibiting the mobility that characterized much of the United States, the people of Appalachia often remained on ancestral land [3]. Due to this isolation from the mainstream, Appalachia became culturally distinct from the rest of the nation [3].

The Appalachian Regional Commission (ARC), created by the United States government to promote economic development in Appalachia, measures county development by comparing three-year unemployment rate, per capita income, and poverty rate with corresponding values for the entire United States [1]. The ARC classifies Appalachian counties as: distressed (worst 10%, when compared to all counties in the nation), at-risk (between the worst 10% and 25%), transitional (between the worst 25% and best 25%), competitive (between the best 25% and 10%), and achievement (best 10%). Locations and numbers of counties in each category can be obtained from Figure 1. In the Appalachian region, 69% of counties are designated as Health Professionals Shortage Areas (HPSAs). More critically, 91% of the distressed counties are HPSAs [4]. This shortage could contribute to people seeking care later in their disease, and might contribute to people not obtaining preventive services that could help them prevent or delay diabetes. These counties often have weak, single-source economies, dominated by coal and tobacco. These economies can be severely disrupted by changing national economic conditions, leaving residents with few options to fall back on when these industries falter [5].

The prevalence of diagnosed diabetes is 9.8% (95% confidence interval [CI]: 9.7, 9.8) for Appalachia and 7.8% (7.8, 7.9) for the rest of the nation (CIs calculated by the authors, not previously published) [6]. The distressed counties have a greater prevalence of diagnosed diabetes than risk profiles (demographic and behavioral factors that are associated with diabetes) alone explain [6]. The unadjusted prevalence of diagnosed diabetes, by county type, is: 13.1% (11.6, 15.0) (distressed), 10.9% (10.0, 11.8) (at-risk), 10.0% (9.4, 10.5) (transitional), 8.8% (7.7, 9.8%) (competitive), 6.3% (5.2, 7.5) (achievement), and 8.2% (8.0, 8.5) (non-Appalachian) [6]. The authors' personal observations suggest that many people living in the poorer counties in Appalachia who have prediabetes often take the condition less seriously than they should (LB) and that a high prevalence of childhood obesity exists, which could lead to early diabetes (RC). Based on these, we speculated that people in the poorer counties who develop diabetes might do so at younger ages than those in non-Appalachian counties. Here, we examine age of diagnosis among people with diagnosed diabetes, both with and without adjusting for risk profile and access to health care at the time of data collection, among counties at different development levels.

## Methods

### Data source

The Behavioral Risk Factor Surveillance System (BRFSS) is a state-based system of repeated cross-sectional health surveys. The BRFSS annually assesses key behavioral risk factors and chronic conditions in noninstitutionalized United States adults 18 years or older. Participants are selected, using random digit dialing, from civilian residents with land-line telephones. We used data from the combined 2006, 2007, and 2008 BRFSS from all states that contain one or more counties that the ARC considered part of Appalachia in 2007. We combined years of data because, for any single data year, the sample sizes in counties at some levels of development were too small for meaningful analysis. Since we combined years, we were restricted to analyses of items included in BRFSS each year between 2006 and 2008. Our data set consisted of 46,355 respondents, both with and without diagnosed diabetes, from Appalachian counties, and 150,679 respondents from non-Appalachian counties in states that contained some part of Appalachia. Data from all people reporting diagnosed diabetes who reside in a state that contains any Appalachian counties were used; because no county-level variables other than level of development were considered, county-level sample sizes did not directly influence the analysis. The number of survey respondents with self-reported diabetes (sample size per level of county development) was: 339 (all distressed counties combined); 839 (all at-risk counties combined); 2,727 (all transitional counties combined); 644 (all competitive counties combined); 101 (all achievement counties combined); and 17,773 (all residents of non-Appalachian counties in states that include some Appalachian counties).

Self-reported diagnosed diabetes status was assessed by the answer to, "Have you ever been told by a doctor that you have diabetes?" Women who reported only having diabetes during pregnancy were not considered to have diabetes. Self-reported age at diagnosis was determined by the question, "How old were you when you were told you have diabetes?" Physical activity was assessed by, "During the past month, other than your regular job, did you participate in any leisure time physical activity?" We defined smoking via, "Have you smoked at least 100 cigarettes in your entire life?" This definition results in current and former smokers being combined. We calculated body mass index (BMI) as self-reported weight (kg) (participants were asked, "About how much do you weigh without shoes?") divided by self-reported height squared (m^2^) (participants were asked, "About how tall are you without shoes?"). Sociodemographics (race/ethnicity, sex, education, and income) were self-reported. Insurance status (did/did not have insurance, of any type, at the time of the survey) and contact with the medical system (did/did not report having a medical visit within the year prior to the survey) were self-reported.

### Analysis

We conducted a person-level analysis, limited to respondents with self-reported diagnosed diabetes. We treated classification of county of residence at the time of survey as an exposure. We used county classifications: distressed, at-risk, transitional, competitive, achievement, and non-Appalachian, as of 2007. The classification "non-Appalachian" refers to the counties outside Appalachia but within the 13 states that included Appalachian counties. We chose these counties as a comparison group to minimize differences due to state policies and programs (e.g., it is unclear that states without Appalachian counties would provide a meaningful comparison). Since demographics (such as educational attainment and income) and behavior (such as smoking, sedentary lifestyle, and being obese) differ among county classifications [6], unadjusted comparison confounds risk profiles with county of residence. Thus, we compared county types both with and without adjusting for selected factors associated with diabetes.

To calculate an adjusted age of diagnosis, we conducted a linear regression, with self-reported age of diagnosis of diabetes as the dependent variable. The independent factors considered were: classification of county of residence; sex; race/ethnicity (non-Hispanic white, non-Hispanic black, multiracial, Hispanic or Latino, and other [where "other" means "all persons not identifying themselves as Hispanic, Latino, black, white, or multiracial"]); education (did not graduate high school, graduated high school, attended but did not complete college or technical school, completed college or technical school); annual household income (< $15000, $15000 to < $25,000, $25000 to < $35,000, $35,000 to < $50,000, ≥$50,000); cigarette smoking status (ever smoked versus never smoked); report of engaging in no leisure-time physical activity in the last month; obesity, defined by BMI ≥30 kg/m^2^; and access to health care. To measure access to health care, we included measures for did/did not have insurance (any type) at the time of the survey and did/did not have at least one medical visit in the year preceding the survey. We also fit the same model without the "access to health care" variables, to determine the impacts of the risk profiles.

As a sensitivity analysis, we ran the regression omitting non-Appalachian counties and using transitional counties (the counties that are economically most like the bulk of United States counties) as the reference group. This was done both because those who specifically study Appalachia might be more interested in a comparison of the poorer counties with the less poor counties, and to counter possible concerns about the comparability of Appalachian counties with counties not in the Appalachian region. We conducted weighted analyses using SUDAAN Version 10.0, accounting for the BRFSS' complex sample design to make the results representative of the states included. We considered results significant if p < 0.05. Data collection for the BRFSS was approved by the Centers for Disease Control and Prevention Institutional Review Board; since this was a secondary analysis of those data, no further review was required.

## Results

Unadjusted average ages at diagnosis for the county types appear in Table 1. An analysis of variance rejected the null hypothesis that the unadjusted mean age of diagnosis is the same in all county types (p < 0.01). Although unadjusted average ages differ significantly, the range is small (50.0 to 52.0 years). An examination of the data suggested that unadjusted self-reported age at diagnosis has a distribution that is reasonably symmetric about its median (results not shown). Table 1 indicates that standard deviations differ little among county types.

**Table 1:** Unadjusted average age at diagnosis

Appalachian Regional Commission county classification	Unadjusted average. age at diagnosis, years (standard deviation of individual age at diagnosis)
Non-Appalachian counties	51.3 (14.7)
Attainment counties	50.5 (15.1)
Competitive counties	51.8 (15.5)
Transitional counties	52.0 (14.8)
At-risk counties	50.9 (14.4)
Distressed counties	50.0 (14.9)

Table 2 displays the coefficients of the linear regression, which allows us to assess impact of development level of county of residence after adjusting for selected factors associated with diabetes and access to care. Positive coefficients indicate later diagnosis of diabetes and negative coefficients indicate earlier diagnosis.

**Table 2:** Linear regression for age at diagnosis of diabetes (years) among those with diagnosed diabetes (N = 22,109)

	Change in average age at time of diagnosis relative to reference level (95% CI)	p-value
Appalachian Regional Commission development category^a^		
Non-Appalachian	Reference	NA
Distressed	-2.8 (-4.7, -1.0)	< 0.01
At-risk	-2.2 (-3.9, -0.4)	0.02
Transitional	0.2 (-0.8, 1.2)	0.65
Competitive	0.7 (-1.4, 2.8)	0.50
Attainment	-0.6 (-3.8, 2.6)	0.71
Sex		
Female	Reference	NA
Male	-0.0 (-0.8, 0.8)	0.98
Annual Income^b^		
≥ $50,000	Reference	NA
$35,000 - < $50,000	2.5 (1.2, 3.7)	< 0.01
$25,000 - < $35,000	4.6 (3.2, 6.1)	< 0.01
$15,000 - < $25,000	5.0 (3.8, 6.3)	< 0.01
< $15,000	2.8 (1.6, 4.2)	< 0.01
Race/ethnicity		
Non-Hispanic white	Reference	NA
Non-Hispanic black	-4.9 (-6.0, -3.9)	< 0.01
Hispanic or Latino	-6.7 (-9.6, -3.8)	< 0.01
Non-Hispanic multiracial	-3.2 (-6.4, 0.1)	0.06
Non-Hispanic other race	-6.1 (-9.1, -3.1)	< 0.01
Education		
Graduated college or technical school	Reference	NA
Attended but did not complete college	-0.6 (-1.8, 0.7)	0.38
Graduated high school	0.6 (-0.6, 1.8)	0.30
Did not graduate high school	2.9 (1.4, 4.3)	< 0.01
Smoking		
No	Reference	NA
Yes	1.6 (0.7, 2.4)	< 0.01
Physical Activity		
Yes	Reference	NA
No	0.9 (0.1, 1.7)	0.03
Obesity		
No	Reference	NA
Yes	-3.1 (-3.9, -2.2)	< 0.01
Any insurance coverage		
Yes	Reference	NA
No	-6.4 (-7.8, -5.0)	< 0.01
Received medical care in the last year		
Yes	Reference	NA
No	-3.7 (-5.0, -2.4)	< 0.01

^a ^The Appalachian Regional Commission classifies Appalachian counties as: distressed (worst 10%, when compared to all counties in the nation), at-risk (between the worst 10% and 25%), transitional (between the worst 25% and best 25%), competitive (between the best 25% and 10%), and achievement (best 10%).

^b ^Income is in dollars as of time of survey.

For those at reference level for all independent factors in the model, the modeled average age at diagnosis was 48.2 years (46.8, 49.5). This is determined from the model intercept. Table 2 indicates that, after adjustment, people with diabetes living in distressed counties, on average, had their diabetes diagnosed 2.8 (1.0, 4.7) years earlier than comparable people living in non-Appalachian counties, and people with diabetes living in at-risk counties, on average, had their diabetes diagnosed 2.2 (0.4, 3.9) years earlier than comparable people living in non-Appalachian counties. Results from the model without the "access to healthcare" variables were similar, with people in distressed counties having diabetes diagnosed 3.1 (1.5, 5.1) years earlier and people in at-risk counties having diabetes diagnosed 2.7 (1.2, 4.2) years earlier than people in non-Appalachian counties. In both analyses, people residing in transitional, competitive, and attainment counties were not significantly different from those living non-Appalachian counties in their age at diagnosis.

Table 2 indicates that obese people and those who are not non-Hispanic white (with the possible exception of multiracial respondents [p = 0.06]) had their diabetes diagnosed earlier. Those with less than a high school education had their diabetes diagnosed later, as did those who had ever regularly smoked at the time of the survey. An annual income of ≥ $50,000 was associated with earlier diagnosis of diabetes. People who reported no leisure-time physical activity had their diabetes diagnosed about a year later than those who did. Sex was not significantly related to age at diagnosis. People with insurance coverage and people who had a health care visit within the last year had their diabetes diagnosed later than those who did not.

For linear regression to be valid, residuals need to be at least approximately normally distributed and have at least approximately constant variance. A visual examination of the residuals from the model indicated that these assumptions were satisfied (results not shown).

In the sensitivity analysis, residence in a distressed or at-risk county remained a significant risk factor for earlier diagnosis (results not shown). In both models, the coefficients associated with counties other than the distressed or at-risk counties were close to zero. Other parameters were roughly similar between the sensitivity analysis and the primary analysis (results not shown).

## Discussion

As far as we are aware, this is the first assessment of age of diagnosis of diabetes in Appalachia. The unadjusted average ages, while statistically significantly different, do not differ substantially. However, an unadjusted comparison confounds risk profile with county development level; because risk profiles differ dramatically among levels of county development (e.g., high poverty and low educational attainment in the distressed counties, low poverty and high educational attainment in the achievement counties [6]), this is not the best comparison. The adjusted comparison gives a better picture of the true differences.

The regression model indicates that residents of distressed and at-risk counties had diabetes diagnosed at younger ages than people with diabetes with similar risk profiles living in non-Appalachian counties. The coefficients for Appalachian counties other than distressed and at-risk, in both the primary and the sensitivity analysis, were near zero. This suggests that, for Appalachian counties other than the distressed and at-risk counties, most if not all of the differences in age at diagnosis is attributable to risk profile; however, in distressed and at-risk counties, people had their disease diagnosed at younger ages than risk profiles alone explain. Similarly, the sensitivity analysis suggests that, for competitive and achievement Appalachian counties, most if not all of the differences in age at diagnosis between these counties and the transitional counties are attributable to risk profile differences; however, in distressed counties, people had their disease diagnosed about three years younger than risk profiles alone explain. Putting this in perspective, the effect on age at diagnosis of residence in a distressed county is roughly comparable to that of obesity.

We have no data concerning the time between developing diabetes and having it diagnosed. However, residents in the distressed and at-risk counties have limited access to care [7], which makes it unlikely that the time between developing diabetes and having it diagnosed in these counties is shorter than it is in the more affluent counties. In addition to cost and transportation, other issues make rapid diagnosis unlikely. Coyne [8] reports that cultural attitudes in Appalachia can be a barrier to obtaining care, including the practice of seeking medical care only as a "last resort" and a distrust of health care providers. A relatively large number of health care providers working in Appalachia are foreign born, and Coyne [8] reports that cultural differences with foreign-born providers and high turnover are other barriers to seeking care. Thus, it is likely that people in distressed and at-risk counties developed diabetes younger than their non-Appalachian counterparts, instead of having developed diabetes at the same age and receiving earlier diagnoses.

People who develop diabetes at younger ages can spend more time with undiagnosed, and therefore untreated, diabetes. At the national level, estimates of the percentage of all diabetes cases that are undiagnosed range from 27% [9] to 32% [10], representing an added burden of disease in distressed and at-risk counties. The prevalence of undiagnosed diabetes in Appalachia could be substantially different from the national level. We are aware of no estimates of the prevalence of undiagnosed diabetes in Appalachia, and so cannot assess how large the additional burden is. Untreated diabetes is associated with greater incidence of complications later in life, such as vision loss [11] and renal and cardiovascular damage [12]. Therefore, residents of the distressed and at-risk counties might be at greater risk of eventually developing complications.

Those with lower incomes and those with only a high school education, on average, tended to have diabetes diagnosed later. People in these conditions are less likely to have access to care [13], which could delay diagnosis. They also might be less aware of health issues and therefore less likely to seek care [14]. People with incomes ≥ $50,000 had their diabetes diagnosed sooner, probably because of greater ease in accessing care.

The US Surgeon General has indicated that tobacco use increases the risk of diabetes [15]. We found that people who reported ever smoking had diabetes diagnosed about two years later than comparable nonsmokers. The reason is unknown. Since the message that smoking is harmful to health is pervasive, one might speculate that people who elect to smoke might be less concerned about health. If so, smokers might be less likely to seek care, possibly resulting in later diagnoses.

Obese people had diabetes diagnosed about three years earlier than the nonobese. Obesity is a well-known risk factor for type 2 diabetes. Therefore, obese people might be screened for diabetes more often than nonobese people. Obesity was measured at the time of interview, and the respondent might or might not have been obese at the time of diagnosis; however, obesity, once developed, often persists [16]. Respondents who have been obese since youth are at risk for developing diabetes at a younger age [16].

The lack of access to full-service grocery stores, which contributes to poor diet, contributes to the high rates of obesity in distressed counties. One study of convenience stores in an Appalachian county found that none carried fresh or frozen green vegetables, low-fat milk, or low-fat cheese [17]. Another study found that Appalachian youth knew what healthy foods were, but ate packaged foods because healthy alternatives were unavailable [18].

People who reported no leisure-time physical activity reported diagnosis of diabetes about a year later than those who did. We are not certain why, because physical activity is a well-known way to prevent or delay type 2 diabetes. One possible reason is that the BRFSS measures leisure-time physical activity. Nonleisure-time physical activity, which was unmeasured, might be substantial in Appalachia, due to agricultural work or employment in the mining industry. The failure to account for nonleisure-time physical activity could bias the results. Also, the BRFSS measures physical activity at the time of the survey. Respondents included in this analysis all had diagnosed diabetes. Their prediagnosis activity status is unknown

We found that racial and ethnic minorities had diabetes diagnosed at earlier ages. This is not consistent with Koopman et al. [19], who found no significant difference among racial and ethnic groups in a 1999-2000 national sample. This could be due to Koopman et al's use of a national (versus subnational) sample, use of 1999-2000 (versus 2006-2008) data, or failure to adjust for income and education. Similarly, a study conducted in Norway found that mean age at the time diagnosis for minority groups was eight to 15 years younger than for other Norwegians [20]. This study's authors speculated that the pathophysiological processes for diabetes started or accelerated earlier in minority groups.

We found that people with insurance coverage at the time of the survey had their diabetes diagnosed about six years later than those who did not, and people who had received medical care in the year preceding the survey had their diabetes diagnosed about four years later than those who did not. These findings are consistent with the notion that access to medical care helps to delay the development of diabetes, and people without access to medical care are likely to develop the disease at younger ages.

Our analysis is subject to several limitations. Data from the BRFSS are self-reported and subject to nonresponse bias, social desirability bias, bias from exclusion of households without land-line telephones, and recall bias. Recall bias concerning the age of diagnosis of diabetes is of particular concern. We can think of no reason that people in the poorer counties would be more likely to incorrectly recall their diabetes as being diagnosed at younger ages than people in other counties, but neither can we totally rule out this possibility. Our measures of access to care were as of the time of the survey. Respondents' insurance status and receipt of medical care might have been different when they developed diabetes or had the condition diagnosed than it was at the time of the survey. We were unable to distinguish type 1 from type 2 diabetes. While physical activity and weight loss can prevent or delay type 2 diabetes, no way of preventing type 1 diabetes is known. However, nationally, type 2 diabetes accounts for between 90% and 95% of all cases of diabetes [7]. Finally, ARC county classifications can change, and we used the classifications as of 2007.

It would have been desirable to provide state, rather than regional, estimates. The number of respondents with diabetes stratified simultaneously by development level and state was too small to support state-level estimates. Thus, our analysis could only be done on a regional level.

## Conclusion

Residents of distressed and at-risk Appalachian counties are at substantial risk for diabetes [6], and are probably at risk of developing it sooner than residents of counties outside Appalachia. Although we could not directly measure this, residents of distressed and at-risk counties in Appalachia are probably at greater risk for complications of diabetes, such as blindness, kidney disease, and lower limb amputation. We cannot prevent diabetes in those that already have the disease. However, we can prevent or delay type 2 diabetes, which accounts for the overwhelming majority of cases nationally, in those who do not already have the disease.

Age, race, and sex, all risk factors for developing diabetes, are not modifiable; education (except possibly for the young) and income are difficult to modify. Physical activity, smoking, and obesity are all modifiable, and thus should be the focus of interventions intended to prevent diabetes. Obesity, lack of physical activity, and smoking, all of which contribute to type 2 diabetes, are common in distressed and at-risk counties [6]. To address this situation, we recommend that residents of distressed and at-risk Appalachian counties be considered a health disparity population. Furthermore, we recommend that states containing Appalachian counties, particularly the distressed and at-risk counties, consider implementing culturally sensitive programs, preferably using community members. These programs should discourage smoking, promote physical activity, encourage a healthy diet, and increase understanding of physical activity and cutting calories as a means of weight loss. Such programs have the potential to eventually decrease the disparity between Appalachia and non-Appalachian regions, both in prevalence of diabetes and age of developing diabetes. To increase the availability of healthy food options, community-based programs that encourage growing fruits and vegetables for home consumption should be considered. States or communities should also consider providing incentives to food retailers to locate in underserved areas and to offer healthier food and beverage choices.

Griffith et al. [21] found that people residing in rural Appalachia perceive themselves as healthy, even if they are obese, lead sedentary lifestyles, and/or have hypertension. Steps should be taken to help people in rural Appalachia realize that people who are obese, lead sedentary lifestyles, and/or have hypertension are not healthy. While many barriers to seeking health care exist, those who believe themselves healthy, regardless of actual health, could be less likely to seek care. Not seeking health care when one has prediabetes could lead to a lack of steps being taken to prevent or delay prediabetes from transitioning to diabetes; the findings that people without insurance or who did not recently seek medical care have their diabetes diagnosed later increase the plausibility of this speculation. Lack of access to health care and lack of seeking healthcare when needed could both contribute to both the increased prevalence and the earlier age at diagnosis in the poorer counties in Appalachia.

To help overcome cultural barriers that are limiting care, steps should be taken to foster community ties and understanding between people in the poorer regions of Appalachia and foreign-born health care providers. Education about the role of medical specialists and the importance of seeking preventive care and screenings may help overcome a cultural preference to seek care only for urgent medical conditions.

Finally, while the steps described above are important, they are not sufficient. To join the economic mainstream, Appalachia needs to move beyond its traditional tobacco- and coal-based economy with its associated "boom and bust" cycle. Improved economic development would likely lead to better health literacy, better access to health care, and an improved quality of health care. The road to achieving these goals might be long and convoluted, but, for the people of Appalachia, it is a necessary step for achieving health equity.

The findings and conclusions in this report are those of the authors and do not necessarily represent the official position of the Centers for Disease Control and Prevention.

## List of Abbreviations

ARC: Appalachian Regional Commission; BRFSS: Behavioral Risk Factor Surveillance System; CI: confidence interval

## Competing interests

The authors declare that they have no competing interests.

## Authors' contributions

LB conceived the study and wrote most of the manuscript, RG did the data analysis, RC and MS reviewed the manuscript and provided guidance on issues related to Appalachian health. All authors read and approved the final manuscript.
